# Nano/Micromotors in Active Matter

**DOI:** 10.3390/mi13020307

**Published:** 2022-02-17

**Authors:** Chenglin Lv, Yuguang Yang, Bo Li

**Affiliations:** 1 Institute of Biomechanics and Medical Engineering, Applied Mechanics Laboratory, Department of Engineering Mechanics, Tsinghua University, Beijing 100084, China; lvcl20@mails.tsinghua.edu.cn; 2 Chemical & Biomolecular Engineering, Johns Hopkins University, Baltimore, MD 21218, USA

**Keywords:** nano/micromotor, cytoskeleton, bacterium, cell, active matter

## Abstract

Nano/micromotors (NMMs) are tiny objects capable of converting energy into mechanical motion. Recently, a wealth of active matter including synthetic colloids, cytoskeletons, bacteria, and cells have been used to construct NMMs. The self-sustained motion of active matter drives NMMs out of equilibrium, giving rise to rich dynamics and patterns. Alongside the spontaneous dynamics, external stimuli such as geometric confinements, light, magnetic field, and chemical potential are also harnessed to control the movements of NMMs, yielding new application paradigms of active matter. Here, we review the recent advances, both experimental and theoretical, in exploring biological NMMs. The unique dynamical features of collective NMMs are focused on, along with some possible applications of these intriguing systems.

## 1. Introduction

From the steam engine invented by Thomas Newcomen in the 18th century to today’s advanced aeroengine, a variety of motors have helped mankind to change the world. Nowadays, alongside macroscopic motors, nano/micromotors (NMMs) have attracted increasing attention due to their important applications and novel functions [1,2]. For example, myosin, dynein and kinesin that are natural NMMs can carry molecular goods along with cytoskeletal fibers [3]. Self-assembled active colloidal systems are artificial NMMs powered by light and can rotate in a liquid crystal [4]. Advances in synthetic biology, nanotechnology, and control strategies have greatly facilitated the creation and manipulation of NMMs.

Recently, active matter has become popular and is a fundamental unit for manufacturing NMMs. In general, active matter belongs to nonequilibrium-condensed systems [5]. Compared with inactive counterparts, active matter can transform ambient or stored energy into movement and drive the whole systems out of equilibrium [6,7]. This property endows active matter systems with rich collective behaviors, which can be harnessed to design and fabricate NMMs.

Common active matter includes animal groups moving collectively, reconstructed cytoskeletal filaments (RCFs), bacterial suspensions, cell tissues and artificial systems from self-propelled colloidal particles to robotic swarms [8,9]. Derived from the cell cortex, the treadmilling process of filaments and interaction between molecular motors and filaments make RCFs active [10]. In bacterial suspensions, bacteria exhibit coupling of random fluctuation and active motion [6]. These self-propelled Brownian particles can drive passive objects to move in suspensions [11] and show directed motion on an asymmetry base [12]. Cell tissues like sperm can also power NMMs [13].

In this review, we focus on NMMs in active matter and discuss several representative types of NMMs. In Section 2, we describe the concept and properties of RCF-based NMMs. We discuss the application of RCF-based NMMs in mimicking cell structure and transporting molecular cargos. In Section 3, we explain the principle of bacterium-based NMMs. Bacterium-driven directed motors and particles transport and separation mediated by bacterium-based NMMs are introduced. In Section 4, we review NMMs consisting of cell tissues such as sperm cells, red blood cells and muscle cells. In Section 5, we introduce the methods to control and regulate NMMs. Finally, we describe the potential applications of these NMMs and discuss the challenges and outlook in this field.

## 2. NMMs Consisted of RCFs

Cells are the basic units of life systems. From movement to proliferation, cells can show complex and diverse dynamic behaviors. The generation of these dynamic behaviors is governed by three types of cytoskeletal filaments in cytoskeleton including microfilaments, intermediate filaments and microtubules (MTs) [14]. There are generally three types of cytoskeleton-based NMMs employing different filaments as tracks to produce movements: myosin motors moves on microfilaments whereas kinesin motors and dynein motors move on MTs [3]. In vitro experiments, when mixing cytoskeletal filaments with cytoskeletal motors, RCF systems can be prepared. Different from the inactive filament structures, RCFs show dynamic structural changes even in thermal equilibrium state. There are two primary mechanisms that underlie the emerging active behaviors. On one hand, the structures of microfilaments and MTs are out of equilibrium. The monomer protein polymerizes at one end of filaments and depolymerizes at the other end [10]. On the other hand, cytoskeletal motors can combine with, move on and detach from cytoskeletal filaments which allow RCFs to be used to manufacture NMMs. MT–kinesin systems and microfilament–myosin systems are common RCF systems.

### 2.1. Classification of the RCF’s Behavior

**Figure 1:**
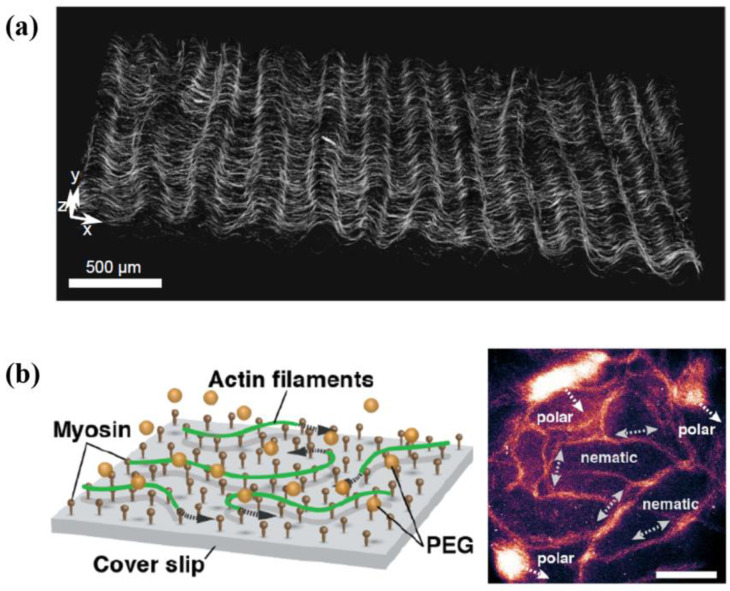
Some behaviors exhibited in reconstructed cytoskeletal filament (RCF) systems. (**a**) A corrugated sheet consists of RCFs in the flow cell. Reprinted with permission from Reference [16], Copyright 2019, National Academy of Sciences. (**b**) The schematic of cytoskeletal filaments gliding experiment and coexistence of polar and nematic phases. Scale bar, 100 µm. Reprinted with permission from Reference [17], Copyright 2018, American Association for the Advancement of Science.

Rich RCF behavior can produce diverse RCF-based NMMs. In virtue of multiple experimental systems such as the oil–water interface [15], flow cell [16] and cover slip [17], RCF systems exhibit a myriad of dynamic behaviors. Because of the asymmetry structure of monomer protein, microfilaments and MTs are polar [18]. In high concentration, these rod-like filaments can arrange to a nematic or polar phase [19]. When assembled at the oil–water interface, RCF systems can form a nematic liquid-crystalline phase with +1/2 and −1/2 defects [15]. The degree of activity and fiber stiffness influence the behaviors [20] and morphology [21] of defects respectively. The intrinsic properties of the system, such as chirality, can affect the dynamic behavior of RCF-based active nematic, presenting complex phenomena such as defect ordering [22,23,24]. Due to the relaxation in the third direction, RCF systems can create a thin corrugated sheet of well-defined wavelength [16,25] (Figure 1a). The wrinkle length is independent of ATP concentration [25] but controlled by the motor concentration [16]. When anchoring cytoskeletal motors on the cover slip, cytoskeletal filaments can glide on the surface powered by motors [17]. Polar and nematic phases can coexist in these systems and switch between each other [17] (Figure 1b). We can exchange the roles of motors and filaments, and cytoskeletal motors will move along the cytoskeletal filaments [26], just like what they do inside cells. These rich dynamic behaviors of RCF-based NMMs have many applications. In the following, we will introduce some representative applications of RCF-based NMMs.

### 2.2. Mimicking Cellular Structures via RCF-Based NMMs

When assembled in droplets or vesicles, RCF-based NMMs can form active bundles [27], nematic layers [15] or cortex-like networks [28,29], which can be leveraged to mimic cellular structures. This type of NMMs exhibits amazingly cell-like activities. Equipped with depletion agents and composite motors, MTs can form cilia-like active bundles at the interface of air bubbles [27]. These MT bundles show self-oscillation, akin to the motion of flagellums of *chlamysomonas*. Composite motors consisting of biotin-labeled kinesin and streptavidin power the entire NMMs. In the right proportions, one molecule of streptavidin can combine with two molecules of biotin-labeled kinesin. Such composite motors can bind and move along two neighboring MTs simultaneously, resulting in relative displacement between them [27] and this property makes composite motors widely used in NMMs consisting of MT–kinesin systems.

As they are organized on the flat surface, the MT–kinesin system exhibits coherent spontaneous flows, which can drive the whole droplet to move [15]. Similarly, when encapsulated into unilamellar vesicles, the microfilament–myosin system can self-organize to cortex-like networks [28,29]. Using RCF-based NMMs to mimic cellular structures contributes to an understanding of the role of structures such as cell cortex and flagellum in cell movement and deformation and can also provide a basis for the making of artificial cells.

### 2.3. RCF-Based NMMs for Self-Organizing Molecular Transport

Radial structures of MTs are very common in cells; for example, radial spindles form during mitosis to separate the chromosomes [30]. In vitro experiments showed that MT–kinesin systems can self-assemble to radial MT asters with the help of central anchored molecules [31] or polymer-stabilized microfluidic droplets [32], which are unique biotemplates for the assembly of NMMs. When combining with MT asters, functionalized composite cytoskeletal motors can carry molecular cargo along MTs to implement specific functionalities. This kind of RCF-based NMMs can be used for a self-organizing molecular transport.

**Figure 2:**
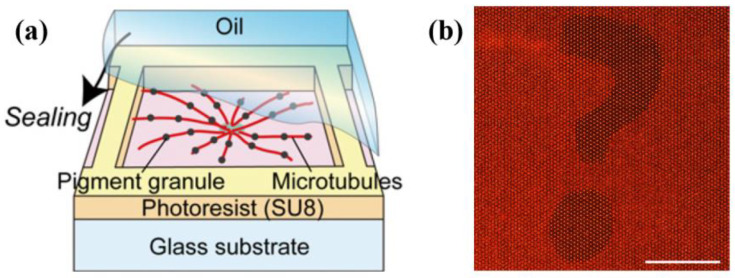
Self-organizing molecular transport system. (**a**) The schematic of the fish melanophore-like optical device. (**b**) A question mask pattern is clearly presented by this artificial optical device. Scale bar, 1 mm. Reprinted with permission from Reference [31], Copyright 2013, National Academy of Sciences.

For example, using NMMs consisting of the MT–kinesin system, a fish melanophore-like optical device can be assembled [31] (Figure 2). Powered by kinesin motors, MTs in the chamber arrange to an aster with minus ends located in the center and plus ends located in the edge. Dynein motors can transport pigment granules along such MT aster to its center, which can change the chamber’s color pattern. This principle is similar to the color-changing mechanism of zebrafish pigment cells. When designed DNA molecules are added, these motors can achieve more complex functions [26]. Specific instructions are encoded in DNA sequences, which can be used to control the assembly and disassembly of MT asters, or to load and unload cargos. A self-organizing molecular transport system makes it possible to fabricate complex functional NMMs.

### 2.4. RCF-Based NMMs as Bioshuttle

**Figure 3:**
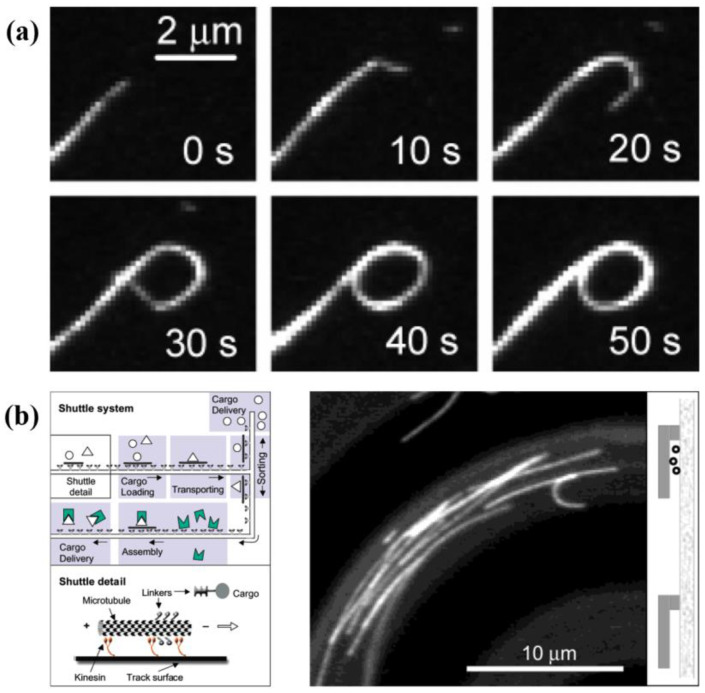
RCF-based bioshuttle. (**a**) Formation of a nanospool structure. Reprinted with permission from Reference [33], Copyright 2005, American Chemical Society. (**b**) The schematic of RCF-based bioshuttle. Reprinted with permission from Reference [34], Copyright 2003, American Chemical Society.

If we attach the motor molecules to the substrate, the cytoskeletal filaments can be pushed to glide along the substrate (Figure 1b). In this experiment system, RCFs can self-assemble into nanowire and nanospool structures [33] (Figure 3a). On a large scale, RCFs can arrange to polar and nematic order states, which can coexist and transition to each other [17]. These interesting phenomena motivate us to modify and optimize such experimental systems to manufacture NMMs, and so bioshuttles come into being [34,35,36,37,38] (Figure 3b). Cytoskeletal filaments are used as shuttles and powered by cytoskeletal motors on the plane or in well-designed micro-tracks. Nitta et al. [35] analyzed the dispersion in active transport by bioshuttles and found that it is comparable to the transport by electroosmotic flow. Protein linkers are attached on cytoskeletal filaments, so RCF-based shuttles can carry cargo molecules [37,38]. Besides carrying goods, this kind of NMM can be used in parallel computation and biosensors [39,40]. By photoetching, combinatorial problems are encoded into the structure of networks. Propelled by molecular motors, cytoskeletal filaments explore the nanostructured network and solve the mathematical problem independently. With the help of bioshuttles, Fischer et al. [40] designed a smart dust biosensor to monitor the environment in organisms or public spaces. In order to design the bioshuttles rationally, Nitta et al. [36] simulated the transport properties of micro-tracks by the Monte–Carlo method. The emergence of bioshuttles explores the potential application of RCF-based NMMs in cargo deliver, biosensors and biocomputation.

## 3. NMMs Consisted of Bacterial Suspensions

Bacteria are a common kind of active particles which achieve active movement through motor organelles such as flagella and cilia [41,42]. In suspensions, bacteria can not only self-organize to form patterns such as bacterial membrane and bacterial chain [43], but also have the property of self-propulsion, which can drive passive particles to a directed motion [6]. These properties of bacteria make them ideal materials for synthesizing or propelling NMMs. In this Section, we review the NMMs composed or powered by bacteria.

### 3.1. Bacterium-Driven Directed NMMs

Back in the 1960s, Feynman pointed out that in nonequilibrium systems, a directed motion can arise from the chaotic dynamics of molecules [44]. Even on asymmetry substrate, Brownian particles show random disordered motion. However, when nonequilibrium conditions such as external periodic drive, particles show spontaneously directed motion which known as ratchet effect [6,45,46]. These unique and directional motions derived from nonequilibrium and asymmetry open the way to assemble bacterium-driven directed NMMs. In this type of NMM, micro-shuttles and micro-gears act as motors, and bacteria propel the whole NMMs by colliding with the motors.

**Figure 4:**
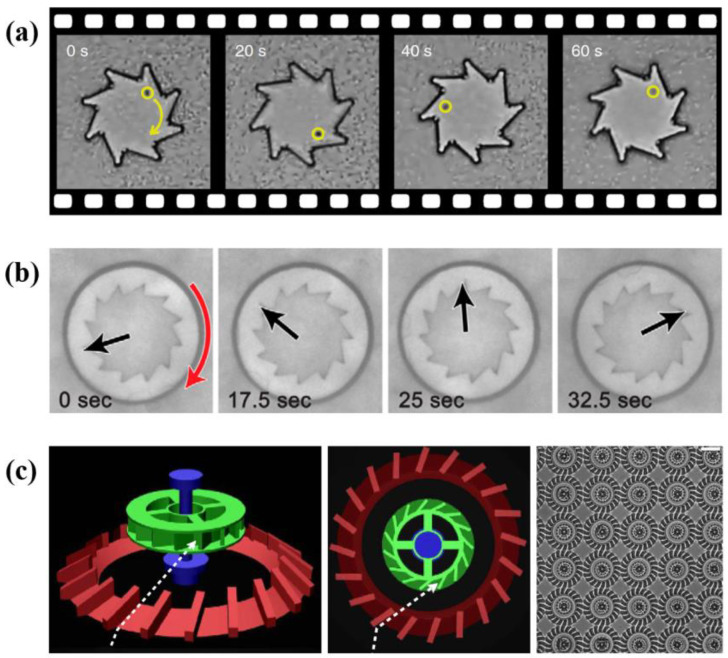
Several types of bacterium-driven directed NMMs. (**a**) Spontaneous rotation of micro-gears in *E. coli* bath. Reprinted with permission from Reference [50], Copyright 2010, National Academy of Sciences. (**b**) Spontaneous rotation of micro-gears with internal teeth in *Bacillus subtilis* bath. Reprinted with permission from Reference [51], Copyright 2010, National Academy of Sciences. (**c**) The structure and array of 3D micro-gears. Scale bar, 20 µm. Reprinted with permission from Reference [52], Copyright 2017, Springer Nature.

Bacterium-driven directed NMMs often display ratchet effect. The breaking in spatial symmetry and time symmetry is essential for supporting directed motion [47]. Through experiments and simulations, arrow-shaped [11] and wedge-shaped [48] micro-shuttles spontaneously perform directed motions in bacterial suspensions. Motion speed shows dependence on the shuttle-shape [11]. Once the shape of the structures is determined, their motion speed increases and then decreases with the bacterial density in the suspensions [48]. This is because, at low density, the more bacteria there are, the greater the co-propelling force is. In the case of high density, things become different. The system shifts into the jamming state, where the more bacteria there are, the less able the object is to move. Besides wedge-like structures, asymmetric structures such as micro-gears can also be used as NMMs in bacterial suspensions [49,50,51]. Angelani et al. [49] numerically proved that it is possible to assemble bacterium-driven directed gears. The key point of this kind of NMMs is trapped bacteria in corners so that bacteria can provide continuous torque for the directional rotation of gears. Di Leonardo et al. [50] and Sokolov et al. [51] fabricated several kinds of chiral micro-gears which can spontaneously rotate in bacterial baths (Figure 4a,b). Vizsnyiczai et al. [52] created more efficient 3D micro-gears (Figure 4c). Ramp structures are designed to guide bacteria into the rotor chamber in the right direction, through which the efficiency and stability of the NMMs are improved obviously. These studies suggest that we can extract energy from bacterial suspensions through well-designed machines, providing promising route towards engineering new NMMs.

### 3.2. Bacterium-Based NMMs Applying to Particles Transport and Separation

In addition to converting energy into directional motion, bacterium-based NMMs can also apply to particles transport. In these NMMs, bacteria act as motors to drive cargo molecules. Some numerical works have investigated the flow of bacteria in channels and the interactions between bacteria and particles [53,54,55]. Based on these simulation, Turiv et al. [56] used streams of bacteria to deliver glass sphere. Nguyen et al. [57] assembled a liposomal bacterium-based NMM to delivery anti-cancer drug. The liposomes loaded with paclitaxel are connected with bacteria via streptavidin–biotin binding mechanism. Driven by bacteria, these NMMs can reach the lesion and release the drugs that target at tumor. With similar binding mechanism, Suh et al. [58] bound poly(lactic-co-glycolic acid) nanoparticles to a tumor-targeting bacterium. They prove that compared with passive diffusing drug particles, these drug delivery NMMs can penetrate deeply in poorly vascularized tumor tissue.

**Figure 5:**
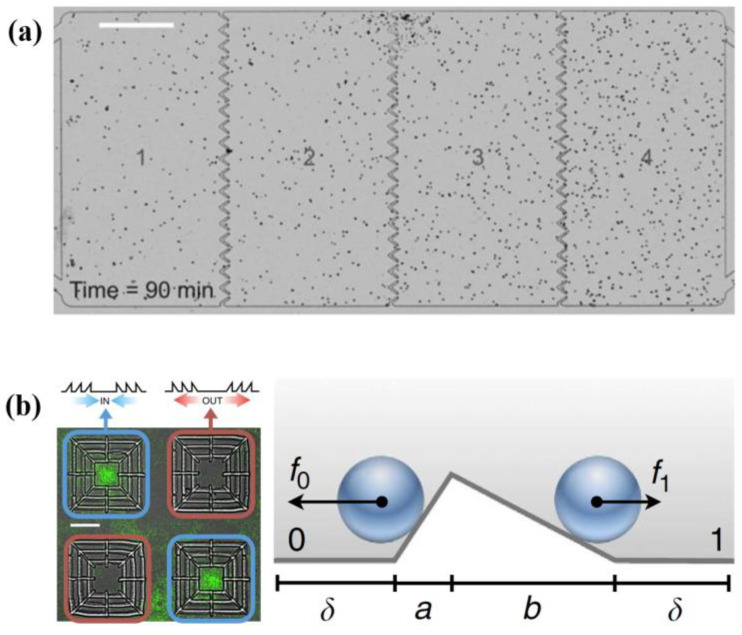
Particles transport and separation mediated by bacterium-based NMMs. (**a**) Funnel-shaped wall used to rectify the bacteria flow. Scale bar, 0.5 mm. Reprinted with permission from Reference [62], Copyright 2013, National Academy of Sciences. (**b**) Particles concentration and depletion driven by bacteria and the schematic of active particles crossing an asymmetric barrier. Scale bar, 20 µm. Reprinted with permission from Reference [12], Copyright 2013, Springer Nature.

Bacterium-based NMMs can also be used in particle separation. Particle separation is a current research hotspot and has been applied in mature fields. For example, Wang et al. [59] prepared anisotropic structural color particles by phase separation of graphene oxide and silica nanoparticles in droplets. These particles are endowed with specific functions and can be used in cell culture, sensing and other aspects. When well-designed barriers are placed in a bacterial bath, particle separation can also occur in this kind of NMM. A wealth of simulations [60,61] and experiments [12,62,63] confirmed that bacteria can be guided to directional motion by microstructures. Funnel-shaped walls in the flow cell can be used to rectify the flow state of initially uniform bacterial suspension [62] (Figure 5a) or to concentrate bacteria [63]. Reichhardt et al. [60] numerically proved that the strength of ratchet effects in these NMMs are dependent on the barrier geometry and the topological property and density of bacteria.

NMMs-mediated particles transport and separation have a wide range of applications. Active particles can be separated in terms of their swimming modes or chirality [6,64,65]. If funnel-shaped walls are arranged along designed structures, the NMM can be used to display specific patterns [63]. Koumakis et al. [12,61] confirmed that bacteria can spontaneously and efficiently deliver and store colloidal beads in designed asymmetry 3D structures. Figure 5b shows the main geometric features of asymmetric barriers. At the same barrier height, if a>b, bacteria can transport beads into the centers of barrier; if a<b, the barrier centers are the forbidden zones of beads. A generalized effective temperature has been introduced to explain the property of this motor [61]. Bacterium-driven directed motors and NMMs-mediated particles transport and separation demonstrate that bacterium-based NMMs have wide applications for fabrication and use in bio-machines.

## 4. NMMs Consisted of Cells

Cells can also be used to manufacture NMMs. On the one hand, cell populations exhibit polarization and alignment [66,67], and the kinetic energy and enstrophy of cell monolayer collective flows obey *q*-Gaussian distribution [68]. These findings shed light on the potential biomedical applications of assembling cell-based NMMs. On the other hand, properties as diverse as self-propulsion and biocompatibility enable the NMMs a wide application prospect. In this Section, we will review NMMs consisted of different types of cells.

### 4.1. NMMs Powered by Sperm Cells

**Figure 6:**
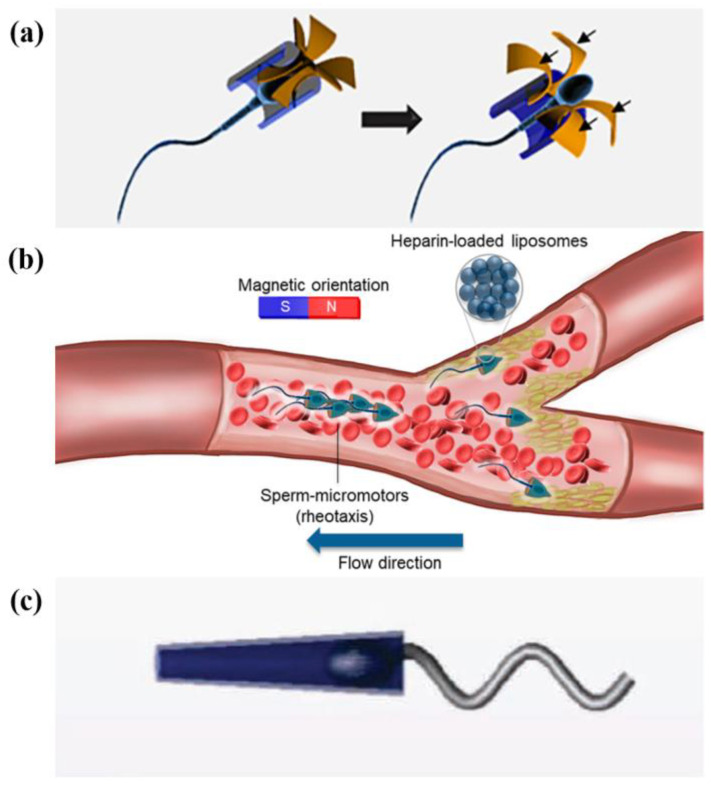
Several types of sperm-powered NMMs. (**a**) Reprinted with permission from Reference [71], Copyright 2018, American Chemical Society. (**b**) Reprinted with permission from Reference [13], Copyright 2020, American Chemical Society. (**c**) Reprinted with permission from Reference [72], Copyright 2013, Wiley-WCH.

Like the bacteria described above, sperm cells are also flexible micro-swimmers, which offers a route to make NMMs. Taking the human sperm cell as an example, it consists of a disc-like head containing genetic information and a long tail which provides a powerful boost to the movement [69]. Biocompatibility, efficient locomotion in the low Reynolds number flow and manipulability make sperm cells become an ideal power source for NMMs [70]. The most common sperm-based NMMs are composed of well-designed microcap and the sperm cell [13,71,72,73,74] (Figure 6). The microcap provides the object for functionalizing the NMM, while the sperm cell serves as the motor for the power. Khalil et al. [73,75] simulated the hydrodynamic effects of NMMs in a low Reynolds number heterogeneous viscous medium and provided theoretical support for the manufacture of NMMs powered by sperm cells.

The design of materials and structures of the microcap will give NMMs specific properties and functions. Magdanz et al. [72] fabricated thin ferromagnetic microtubes and coupled them to bull spermatozoa (Figure 6c). An external magnet can direct the motion of these NMMs. Xu et al. [71] designed a tetrapod ferromagnetic microstructure, and the sperm cell loaded with anticancer drugs pushed the microstructure to move along the magnetic field orientation (Figure 6a). When encountering the tumor cells, the microstructure deforms and allows the sperm cell to be released. The sperm cell can fuse with the tumor cell and delivery drugs, thus achieving the effect of targeted therapy. The high propulsion force enables sperm cells to power NMMs to swim against flowing blood and to deliver cargos [13] (Figure 6b). In various physiological environments, microcaps can protect against oxidative stress that may damage sperm cells [74]. Together, NMMs driven by sperm cells have a good prospect in cargo delivery and targeted therapy and have become a potential treatment for gynecological diseases and cancer.

### 4.2. NMMs Consisted of Somatic Cells

Somatic cells are collections of all cells except for germ cells. Somatic cells can also apply for manufacturing NMMs. Herein, we mainly introduce the NMMs composed of red blood cells (RBCs) and cardiomyocytes.

**Figure 7:**
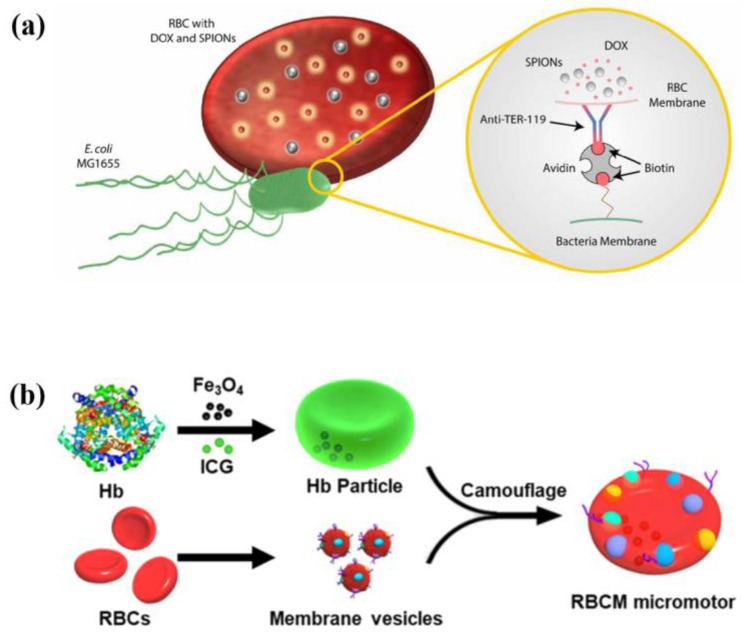
RBC-based NMMs. (**a**) The schematic of bacterium-propelled biohybrid NMMs. Reprinted with permission from Reference [77], Copyright 2018, American Association for the Advancement of Science. (**b**) The schematic of RBC-based NMMs loaded with cargos and magnetic nanoparticles. Reprinted with permission from Reference [78], Copyright 2019, American Chemical Society.

RBCs are natural molecular vehicles in the body. The typical shape of mammals’ RBCs is the biconcave disk. When maturing, RBCs of mammals lose nuclei and all other cellular organelles that improve cargo and deformation capacities [76]. According to the propulsion mode, RBC-based NMMs can be divided into self-propulsion NMMs [77] and field-driven NMMs [78,79,80]. For self-propulsion RBC-based NMMs, RBCs are assembled with bacteria into hybrid NMMs, in which bacteria provide propulsion force and RBCs are responsible for loading cargos. For field-driven RBC-based NMMs, the outfield drive is realized by loading specific particles in RBCs. Alapan et al. [77] designed bacterium-propelled biohybrid NMMs (Figure 7a). RBCs loaded with anticancer drug molecules and magnetic nanoparticles are bound with bacteria, which provide autonomous propulsion for NMMs. Dreyfus et al. [79] assembled an artificial flagellum to drive RBC-based NMMs. The magnetic particles are coated with streptavidin and particles are linked with double-stranded DNA with biotin at each end. A uniform magnetic field is used to align these flagella and another orthogonal magnetic field can drive flagella to oscillate to produce the driving force. Wu et al. [80] and Gao et al. [78] encapsulated molecule cargos and magnetic nanoparticles into RBC-based NMMs (Figure 7b). These NMMs can move spontaneously in ultrasonic field and the direction of motion is controlled by magnetic field. Guo et al. [81] rebuilt RBCs that mimic the properties of native RBCs. Via loading ATP biosensors in rebuild RBCs, this NMM can be used to sense toxin. Due to biocompatibility and ability to travel throughout the body with the circulatory system, RBC-based NMMs are ideal systems for drug delivery.

Muscle cells can also power NMMs because of the ability of powerful contraction [82,83,84,85]. Williams et al. [82] engineered self-propelled sperm-like NMMs. These NMMs are composed of polydimethylsiloxane filaments, which are akin to the sperm cells in shape. Cardiomyocytes are cultured on the tail. The contraction of cardiomyocytes drives the periodic deformation of filaments to power the NMM movement. Guided by bionics and optogenetics, Park et al. [83] created a stingray-like artificial animal. The architecture of this NMM includes a golden skeleton, a muscle layer and two elastomer layers. Through muscle circuits, light stimulation can be used to activate muscle to drive the whole NMM. Inspired by the color changing mechanism of chameleons, Fu et al. [84] developed living structural color hydrogels. Cardiomyocytes drive the periodic changes of inverse opal structure to achieve self-regulation. Chen et al. [85] found that when cultured on *Morpho* butterfly wings, cardiomyocytes recovered their autonomous beating ability through cell orientation and good contractility. They used cardiomyocytes to cause the wings to periodically deform, giving rise to the corresponding structural color. Similar self-powered hybrid NMMs have also been used to make bionic fish [86] and caterpillar-like soft robots [87]. These NMMs, with the help of bionics, not only reveal the mechanisms of the corresponding life processes, but also explore technical routes for the construction of intelligent actuators and soft robots.

## 5. Regulation and Control of NMMs

For motors in daily life, we control them by encoding in order to function better. Likewise, we are eager to stimulate the potential of NMMs based on active matter. In this Section, we review a variety of methods that are used to regulate and control the NMMs.

### 5.1. Physical Field

Although NMMs are capable of converting stored energy to mechanical movement, most of them still require external factors to accomplish a more controllable motion [88]. External physical fields as diverse as light and magnetic field are widely used to regulate and control the behaviors of NMMs.

#### 5.1.1. Light

In the 1980s, Jean-Marie Lehn proposed the light-coupled transport process [89], which is the source of light-regulated NMMs [88]. In recent years, with the rapid development of optogenetics and photocatalysis, the light source has been explored in-depth to regulate the behaviors of NMMs based on active matter [31,52,83,90,91].

**Figure 8:**
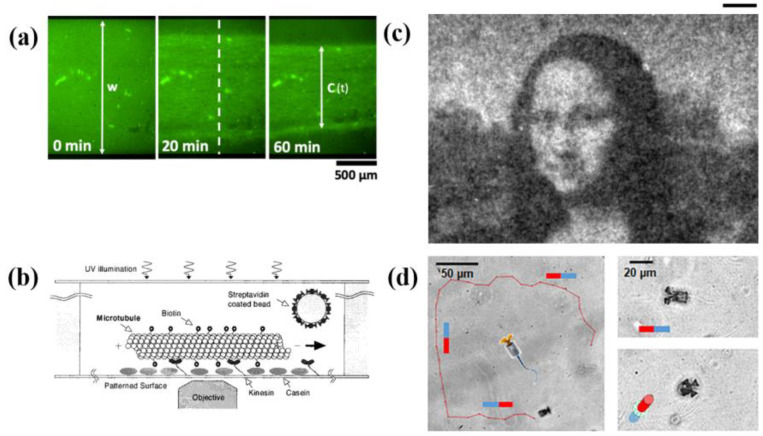
Using physical field to control and regulate active matter-based NMMs. (**a**) Photostimulated contraction of RCFs. Reprinted with permission from Reference [95], Copyright 2021, American Chemical Society. (**b**) The schematic of light-controlled bioshuttle. Reprinted with permission from Reference [91], Copyright 2001, American Chemical Society. (**c**) Mona Lisa pattern shown by light-controlled bacteria system. Scale bar, 100 µm. Reprinted with permission from Reference [98], Copyright 2018, eLife Sciences Publications. (**d**) Track of sperm cell-based NMM controlled by magnetic field. Reprinted with permission from Reference [71], Copyright 2018, American Chemical Society.

Introducing light-activated proteins into the NMM systems is one of the control routes. Ross et al. [92] achieved light-controlled formation, fusion and motion of MT asters via synthetic motors assembled by fusing kinesin motors to optically dimerizable iLID proteins. When exposed to light, the synthetic motors form dimers that can bind and move along two neighboring MTs. Zhang et al. [93] used light-sensitive myosin motors to control the movement of defects. Qu et al. [94] proposed a similar method to control the flow field. Ahamd et al. [95] synthesized light-driven energy vesicles containing bacteriorhodopsin, a light-driven proton pump that can actively transport proton across the cell membrane [96] and EF_0_F_1_-ATP synthase, which can use proton gradient to synthesize ATP. They adopted light-powered vesicles to control the beating frequency of flagella and the contraction of MT networks (Figure 8a).

Activation and deactivation of light-controlled chemical material release are another approach to control and regulate NMMs. This method is common in inactive matter-based NMMs, represented by tiny spheres coated with specific chemicals [97]. Shao et al. [90] introduced this approach in RBC-based NMMs. With the metal sputter-coating method, they produced Janus polymeric particles half covered with gold. In order to move efficiently in the biological environment, erythrocyte membranes are coated on these particles. When exposed to the light, a thermal gradient is generated due to asymmetric distribution of Au, which makes it possible to drive these NMMs to move. Hess et al. [91] controlled the motion of bioshuttle via UV-induced release of caged ATP (Figure 8b). The artificial optical device is activated in a similar way [31].

Light can control the motion direction and velocity of NMMs. Park et al. [83] used the relative intensity among light sources to guide the stingray-like NMM maneuver through constraints. Vizsnyiczai et al. [52] controlled the rotational speed of bacterium-based NMM via light intensities. They designed *E. coli* bacteria, which express bacteriorhodopsin on the membrane. The bacteria can move continuously under the light source, achieving continuous operation of NMMs. The rotational speed can be modulated by adjusting the speed of bacteria and by changing the light intensity. Light can also control the density of bacteria to display patterns. Frangipane et al. [98] introduced genetically modified bacteria, whose motion speeds are controlled by light. They display patterns by changing the density of bacteria in local areas, and can switch between different patterns (Figure 8c).

#### 5.1.2. Magnetic Field

Since Dreyfus et al. [79] assembled magnetically driven and controlled NMMs, magnetic fields have been widely used in the control of NMMs. For different active matter-based NMMs, the implementation of magnetic control is different. For sperm cell-based NMMs, introducing ferromagnetic structure is the main way to realize magnetic control [13,71,72]. Sperm cells can produce powerful thrusts, while ferromagnetic microcaps surrounding their heads regulate the direction of the movement in the magnetic field (Figure 8d). Guided by a magnetic field, sperm cell-based NMMs can navigate through complex passageways to the desired location. For RBC-based NMMs, magnetic control can be achieved by wrapping magnetic nanoparticles or magnetic hemoglobin in RBCs [78,80]. The existence of magnetic nanoparticles also makes it possible for magnetic resonance imaging. Magnetic control shows great potential in RCF-based NMMs. At the oil–water interface experiments, Guillamat et al. [99,100] introduced octyl-cyanobiphenyl(8CB) molecules in the oil layer. 8CB molecules align with the direction of the magnetic field. In orthogonal magnetic field, topological defects can be arranged along the magnetic field [99]. Under the rotating magnetic field, some novel defect structures are generated in active nematic swirls [100]. Zhang et al. [101] explored the general mechanism of pattern synthesis and control in the active system using an electromagnetic field to regulate the active time. In order to improve the application value of magnetic control, more effects need to be realized in the accurate fabrication methods and the make of degradable magnetic materials.

### 5.2. Topological Constraint

In recent years, the effects of topological constraint on active matters have attracted extensive attention. Sipos et al. [102] showed that bacteria can swim along convex wall for a long time, which is called wall entrapment behavior. Bianchi et al. [103,104] studied the mechanism underlying wall entrapment in bacteria suspensions. Experiments and numerical studies showed that the size, topological structure and anchoring properties of constraints have marked influences on RCF systems [105,106,107]. Motivated by these behaviors, topological constraints become a method to regulate and control the behaviors of NMMs. As mentioned before, the ratchet effect promotes the emergence of bacterium-driven NMMs [50,51]. Mahmud et al. [108] used micropatterned ratchets to control the cell motions. They realized non-movement, random movement and directional movement through adjusting the shape of the micro-structure. Hess et al. [109] introduced a ratchet effect in RCFs. In pinwheel patterns, MT-based shuttles can be sorted by the motion direction. Doot et al. [110] generated RCF networks in ratchet channels. Motors can move along the networks to transport cargos directly.

Topological constraints can control the motion of RCFs and regulate the assembly of self-organizing structures [111,112,113]. Clemmens et al. [111] designed a complex track including reflector function, crossing function and circular concentrator to sort and collect bioshuttles. Inoue et al. [112] found that RCFs can form dynamic adaptive patterns according to constraints. Thijssen et al. [113] controlled the motion and number of topological defects in active nematic flow by submersed micropatterns. Clemmens et al. [114,115] analyzed the principles of MTs guiding in the microchannels. They demonstrated that the edges combine both topography and chemistry to attain the highest guiding efficiency, which provides theoretical support for the design of bioshuttles.

**Figure 9:**
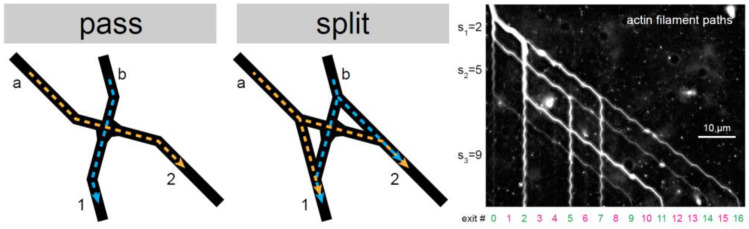
The schematic of behaviors of RCFs in a pass junction and a split junction and the results of parallel computation mediated by actin filaments. Reprinted with permission from Reference [39], Copyright 2016, National Academy of Sciences.

The design and arrangement of constraints enable NMMs to achieve a variety of functions. Nicolau Jr. et al. [39] designed a pass junction and a split junction to guide the motion of actin filaments and MTs. By properly arranging these microstructures, RCF-based parallel computation can be realized (Figure 9). Saper et al. [116] adopted kinesin motors and constraints to accelerate the assembly of MTs. Hess et al. [117] merge the images of MTs gliding on the substrate to explore the surface properties. A piconewton forcemeter is assembled with the RCF-based NMM [118]. These brilliant ideas open up the possibility of building smart nanorobots in the future.

### 5.3. Chemical Field

Chemical field can also control and regulate NMMs in active matter. Most active matter-based NMMs require fuel consumption during operation, so the movement of these NMMs can be controlled by tuning the level of chemical substances such as ATP and oxygen [119]. Sokolv et al. [51] regulated the rotation speed of bacterium-driven gears by tuning the amount of oxygen available to the bacteria. The chemical gradient field can be used to control the direction of the NMMs’ movement. Using chemotaxis, bacterium-based NMMs can induce deflection or directional motion under specific substance concentration gradients [120,121,122]. Singh et al. [123] explored the use of concentration gradient-guided bacterium-based NMMs to deliver anticancer drugs. A glucose gradient is created on the Transwell membrane, and bacterium-based NMMs move towards cancer cells under the guidance of glucose concentration gradient. Other chemicals can also affect the movement of NMM. Liu et al. [124] designed logic gates through multilink 3D printing of bacteria. Three kinds of logic gates including AND, OR and NAND can be obtained by changing the biochemical molecular compositions in the hydrogel at the inputs. Keya et al. [125,126] controlled the collective behavior of RCF-based NMMs via introducing DNA molecules into the systems. They devised photoresponsive DNA to control the NMM switching between solitary and swarm. Genetic engineering has expanded the application of biochemical molecular control. Curatolo et al. [127] introduce engineered *E. coli* to realize complex dynamic behaviors such as phase separation, oscillation and co-location of different populations.

### 5.4. Algorithmic Navigation and Control

The experimental realization of various control strategies paves the way for NMMs to serve as micro/nanoscopic robots that are capable of performing instructed tasks in complex micro-environments. Among the various potential real-world applications ranging from drug delivery to precision surgery to environmental remediation, one fundamental task is to navigate an NMM between two points in an environment. Challenges arising from this task include long-distance travel on the scale magnitudes larger than an NMM, unknown or dynamically changing environment with obstacles and dead-ends. There have been efforts directed towards developing various navigation strategies, including decision rules derived from heuristics [128,129], from the Markov decision process framework [130], as well as from data-driven learning-based methods [131,132]. Particularly, state-of-the-art learning-based method leverages the learning capacity and generalizability of deep neural networks to derive decision rules that enables robots to navigate in unknown environments. Beyond single NMM, collectively controlled NMM swarms can form intelligent systems that carry out non-trivial tasks such as cargo capture and transport in micro-structured environments [133,134].

## 6. Conclusions

In this review, we introduce three kinds of NMMs consisting of active matter including RCF-based NMMs, bacterium-based NMMs and cell-based NMMs. We also discuss the methods to control and regulate NMMs, including physical field, topological constraint, and chemical field, as well as algorithmic control and navigation. Biocompatibility, robustness, and ease of handling make active matter-based NMMs, promising applications. Cargo delivery is one of these applications. Active matter-based NMMs can be guided by the field or self-controlled to carry goods to designated areas. The propulsive force even allows these NMMs to penetrate the membrane structures of the cell and deliver cargos to the interior. Biomedical therapy is another application. At the cellular scale, cargo delivery application allows active matter-based NMMs to acquire targeted drug delivery capability, which can improve the therapeutic efficiency. At the tissue level, these NMMs can be applied to wound healing, thrombolysis and tissue imaging. Environmental detection and nano/micro sensing are also potential applications of active matter-based NMMs. Algorithmic control and navigation address controller design challenges when employing these NMMs as futuristic robots at the microscopic scale.

Despite great progress, active matter-based NMMs still face many challenges before they can enter daily life. First, the process of preparing NMMs in large quantities needs to be explored. In this process, quality control of the products as well as production costs need to be considered. Second, current functions are relatively simple and unitary. How to assign complex functionality to active matter-based NMMs is a problem when it comes to solving real problems. Third, developers must demonstrate the efficiency and reliability of the active matter-based NMMs before it can be put to use.

In summary, active matter-based NMMs have a wide application prospect. The research and development of NMMs provide technological paths for the manufacture of nanorobots, bio-machines and even artificial life. The 1966 movie *Fantastic Voyage* depicts the future of nanomachines: astronauts perform brain surgery in a tiny submarine that penetrates into a patient’s bloodstream [135]. Perhaps in the near future, a similar scenario will appear in life, with the slight difference that doctors will control the active matter-based NMMs to be implemented.

## Data Availability

Not applicable.

## References

[1] Li J., Rozen I., Wang J. (2016). Rocket Science at the Nanoscale. ACS Nano.

[2] Sánchez S., Soler L., Katuri J. (2015). Chemically Powered Micro- and Nanomotors. Angew. Chem. Int. Ed..

[3] Woehlke G., Schliwa M. (2000). Walking on two heads: The many talents of kinesin. Nat. Rev. Mol. Cell Biol..

[4] Yuan Y., Abuhaimed G.N., Liu Q., Smalyukh I.I. (2018). Self-assembled nematic colloidal motors powered by light. Nat. Commun..

[5] Marchetti M.C., Joanny J.F., Ramaswamy S., Liverpool T.B., Prost J., Rao M., Simha R.A. (2013). Hydrodynamics of soft active matter. Rev. Mod. Phys..

[6] Bechinger C., Di Leonardo R., Löwen H., Reichhardt C., Volpe G., Volpe G. (2016). Active particles in complex and crowded environments. Rev. Mod. Phys..

[7] Ramaswamy S. (2010). The Mechanics and Statistics of Active Matter. Annu. Rev. Condens. Matter Phys..

[8] Chaté H. (2020). Dry Aligning Dilute Active Matter. Annu. Rev. Condens. Matter Phys..

[9] Doostmohammadi A., Ignes-Mullol J., Yeomans J.M., Sagues F. (2018). Active nematics. Nat. Commun..

[10] Prost J., Julicher F., Joanny J.-F. (2015). Active gel physics. Nat. Phys..

[11] Angelani L., Di Leonardo R. (2010). Geometrically biased random walks in bacteria-driven micro-shuttles. New J. Phys..

[12] Koumakis N., Lepore A., Maggi C., Di Leonardo R. (2013). Targeted delivery of colloids by swimming bacteria. Nat. Commun..

[13] Xu H., Medina-Sánchez M., Maitz M.F., Werner C., Schmidt O.G. (2020). Sperm micromotors for cargo delivery through flowing blood. ACS Nano.

[14] Fletcher D.A., Mullins D. (2010). Cell mechanics and the cytoskeleton. Nature.

[15] Sanchez T., Chen D.T.N., DeCamp S.J., Heymann M., Dogic Z. (2012). Spontaneous motion in hierarchically assembled active matter. Nature.

[16] Senoussi A., Kashida S., Voituriez R., Galas J.-C., Maitra A., Estevez-Torres A. (2019). Tunable corrugated patterns in an active nematic sheet. Proc. Natl. Acad. Sci. USA.

[17] Huber L., Suzuki R., Krüger T., Frey E., Bausch A.R. (2018). Emergence of coexisting ordered states in active matter systems. Science.

[18] Amos L., Klug A. (1974). Arrangement of subunits in flagellar microtubules. J. Cell Sci..

[19] Zhang R., Mozaffari A., de Pablo J.J. (2021). Autonomous materials systems from active liquid crystals. Nat. Rev. Mater..

[20] Giomi L., Bowick M.J., Ma X., Marchetti M.C. (2013). Defect annihilation and proliferation in active nematics. Phys. Rev. Lett..

[21] Zhang R., Kumar N., Ross J.L., Gardel M.L., de Pablo J.J. (2018). Interplay of structure, elasticity, and dynamics in actin-based nematic materials. Proc. Natl. Acad. Sci. USA.

[22] Li Z.-Y., Zhang D.-Q., Lin S.-Z., Li B. (2020). Pattern formation and defect ordering in active chiral nematics. Phys. Rev. Lett..

[23] Ventejou B., Chaté H., Montagne R., Shi X.-Q. (2021). Susceptibility of orientationally ordered active matter to chirality disorder. Phys. Rev. Lett..

[24] Yang Q., Zhu H., Liu P., Liu R., Shi Q., Chen K., Zheng N., Ye F., Yang M. (2021). Topologically protected transport of cargo in a chiral active fluid aided by odd-viscosity-enhanced depletion interactions. Phys. Rev. Lett..

[25] Strübing T., Khosravanizadeh A., Vilfan A., Bodenschatz E., Golestanian R., Guido I. (2020). Wrinkling instability in 3D active nematics. Nano Lett..

[26] Wollman A.J.M., Sanchez-Cano C., Carstairs H.M.J., Cross R.A., Turberfield A.J. (2014). Transport and self-organization across different length scales powered by motor proteins and programmed by DNA. Nat. Nanotechnol..

[27] Sanchez T., Welch D., Nicastro D., Dogic Z. (2011). Cilia-like beating of active microtubule bundles. Science.

[28] Loiseau E., Schneider J.A.M., Keber F.C., Pelzl C., Massiera G., Salbreux G., Bausch A.R. (2016). Shape remodeling and blebbing of active cytoskeletal vesicles. Sci. Adv..

[29] Dürre K., Keber F.C., Bleicher P., Brauns F., Cyron C.J., Faix J., Bausch A.R. (2018). Capping protein-controlled actin polymerization shapes lipid membranes. Nat. Commun..

[30] Hess H., Ross J.L. (2017). Non-equilibrium assembly of microtubules: From molecules to autonomous chemical robots. Chem. Soc. Rev..

[31] Aoyama S., Shimoike M., Hiratsuka Y. (2013). Self-organized optical device driven by motor proteins. Proc. Natl. Acad. Sci. USA.

[32] Juniper M.P.N., Weiss M., Platzman I., Spatz J.P., Surrey T. (2018). Spherical network contraction forms microtubule asters in confinement. Soft Matter.

[33] Hess H., Clemmens J., Brunner C., Doot R., Luna S., Ernst K.H., Vogel V. (2005). Molecular self-assembly of “nanowires” and “nanospools” using active transport. Nano Lett..

[34] Hess H., Matzke C.M., Doot R.K., Clemmens J., Bachand G.D., Bunker B.C., Vogel V. (2003). Molecular shuttles operating undercover: A new photolithographic approach for the fabrication of structured surfaces supporting directed motility. Nano Lett..

[35] Nitta T., Hess H. (2005). Dispersion in active transport by kinesin-powered molecular shuttles. Nano Lett..

[36] Nitta T., Tanahashi A., Hirano M., Hess H. (2006). Simulating molecular shuttle movements: Towards computer-aided design of nanoscale transport systems. Lab Chip.

[37] Bachand G.D., Rivera S.B., Carroll-Portillo A., Hess H., Bachand M. (2006). Active capture and transport of virus particles using a biomolecular motor-driven, nanoscale antibody sandwich assay. Small.

[38] Ramachandran S., Ernst K.-H., Bachand G.D., Vogel V., Hess H. (2006). Selective loading of kinesin-powered molecular shuttles with protein cargo and its application to biosensing. Small.

[39] Nicolau D.V., Lard M., Korten T., van Delftf F.C.M.J.M., Persson M., Bengtsson E., Månsson A., Diez S., Linke H., Nicolau D.V. (2016). Parallel computation with molecular-motor-propelled agents in nanofabricated networks. Proc. Natl. Acad. Sci. USA.

[40] Fischer T., Agarwal A., Hess H. (2009). A smart dust biosensor powered by kinesin motors. Nat. Nanotechnol..

[41] Berg H.C. (2008). Bacterial flagellar motor. Curr. Biol..

[42] Gibbons I.R. (1981). Cilia and flagella of eukaryotes. J. Cell Biol..

[43] Liu Y., Li B., Feng X.-Q. (2020). Buckling of growing bacterial chains. J. Mech. Phys. Solids.

[44] Feynman R.P., Leighton R.B., Sands M. (1963). The Feynman Lectures on Physics. Mainly Mechanics, Radiaion and Heat.

[45] Astumian R.D. (1997). Thermodynamics and kinetics of a brownian motor. Science.

[46] Reimann P. (2002). Brownian motors: Noisy transport far from equilibrium. Phys. Rep..

[47] Angelani L., Costanzo A., Di Leonardo R. (2011). Active ratchets. Eur. Phys. Lett..

[48] Kaiser A., Peshkov A., Sokolov A., Hagen B.T., Löwen H., Aranson I.S. (2014). Transport powered by bacterial turbulence. Phys. Rev. Lett..

[49] Angelani L., Di Leonardo R., Ruocco G. (2009). Self-starting micromotors in a bacterial bath. Phys. Rev. Lett..

[50] Di Leonardo R., Angelani L., Dell’Arciprete D., Ruocco G., Iebba V., Schippa S., Conte M.P., Mecarini F., De Angelis F., Di Fabrizio E. (2010). Bacterial ratchet motors. Proc. Natl. Acad. Sci. USA.

[51] Sokolov A., Apodaca M.M., Grzybowski B.A., Aranson I.S. (2010). Swimming bacteria power microscopic gears. Proc. Natl. Acad. Sci. USA.

[52] Vizsnyiczai G., Frangipane G., Maggi C., Saglimbeni F., Bianchi S., Di Leonardo R. (2017). Light controlled 3D micromotors powered by bacteria. Nat. Commun..

[53] Angelani L., Maggi C., Bernardini M.L., Rizzo A., Di Leonardo R. (2011). Effective interactions between colloidal particles suspended in a bath of swimming cells. Phys. Rev. Lett..

[54] Costanzo A., Di Leonardo R., Ruocco G., Angelani L. (2012). Transport of self-propelling bacteria in micro-channel flow. J. Phys. Condens. Matter.

[55] Paoluzzi M., Di Leonardo R., Angelani L. (2015). Self-sustained density oscillations of swimming bacteria confined in microchambers. Phys. Rev. Lett..

[56] Turiv T., Koizumi R., Thijssen K., Genkin M.M., Yu H., Peng C., Wei Q.-H., Yeomans J.M., Aranson I.S., Doostmohammadi A. (2020). Polar jets of swimming bacteria condensed by a patterned liquid crystal. Nat. Phys..

[57] Du Nguyen V., Han J.-W., Choi Y.J., Cho S., Zheng S., Ko S.Y., Park J.-O., Park S. (2016). Active tumor-therapeutic liposomal bacteriobot combining a drug (paclitaxel)-encapsulated liposome with targeting bacteria (*Salmonella typhimurium*). Sens. Actuators B Chem..

[58] Suh S., Jo A., Traore M.A., Zhan Y., Coutermarsh-Ott S.L., Ringel-Scaia V.M., Allen I.C., Davis R.M., Behkam B. (2019). Nanoscale bacteria-enabled autonomous drug delivery system (NanoBEADS) enhances intratumoral transport of nanomedicine. Adv. Sci..

[59] Wang H., Liu Y., Chen Z., Sun L., Zhao Y. (2020). Anisotropic structural color particles from colloidal phase separation. Sci. Adv..

[60] Reichhardt C., Reichhardt C.J.O. (2013). Active matter ratchets with an external drift. Phys. Rev. E.

[61] Koumakis N., Maggi C., Di Leonardo R. (2014). Directed transport of active particles over asymmetric energy barriers. Soft Matter.

[62] Kantsler V., Dunkel J., Polin M., Goldstein R.E. (2013). Ciliary contact interactions dominate surface scattering of swimming eukaryotes. Proc. Natl. Acad. Sci. USA.

[63] Galajda P., Keymer J., Chaikin P., Austin R. (2007). A wall of funnels concentrates swimming bacteria. J. Bacteriol..

[64] Volpe G., Buttinoni I., Vogt D., Kümmerer H.-J., Bechinger C. (2011). Microswimmers in patterned environments. Soft Matter.

[65] Mijalkov M., Volpe G. (2013). Sorting of chiral microswimmers. Soft Matter.

[66] Lv J.-Q., Chen P.-C., Guan L.-Y., Góźdź W.T., Feng X.-Q., Li B. (2021). Collective migrations in an epithelial–cancerous cell monolayer. Acta Mech. Sin.-PRC.

[67] He S., Green Y., Saeidi N., Li X., Fredberg J.J., Ji B., Pismen L.M. (2020). A theoretical model of collective cell polarization and alignment. J. Mech. Phys. Solids.

[68] Lin S.-Z., Zhang W.-Y., Bi D., Li B., Feng X.-Q. (2021). Energetics of mesoscale cell turbulence in two-dimensional monolayers. Commun. Phys..

[69] Smith D.J., Gaffney E.A., Blake J.R., Kirkman-Brown J.C. (2009). Human sperm accumulation near surfaces: A simulation study. J. Fluid Mech..

[70] Singh A.V., Ansari M.H.D., Mahajan M., Srivastava S., Kashyap S., Dwivedi P., Pandit V., Katha U. (2020). Sperm Cell Driven Microrobots—Emerging Opportunities and Challenges for Biologically Inspired Robotic Design. Micromachines.

[71] Xu H., Medina-Sánchez M., Magdanz V., Schwarz L., Hebenstreit F., Schmidt O.G. (2018). Sperm-hybrid micromotor for targeted drug delivery. ACS Nano.

[72] Magdanz V., Sanchez S., Schmidt O.G. (2013). Development of a sperm-flagella driven micro-bio-robot. Adv. Mater..

[73] Khalil I.S.M., Klingner A., Magdanz V., Striggow F., Medina-Sánchez M., Schmidt O.G., Misra S. (2019). Modeling of spermbots in a viscous colloidal suspension. Adv. Theory Simul..

[74] Ridzewski C., Li M., Dong B., Magdanz V. (2020). Gelatin microcartridges for onboard activation and antioxidant protection of sperm. ACS Appl. Bio Mater..

[75] Khalil I.S.M., Klingner A., Hamed Y., Magdanz V., Toubar M., Misra S. (2019). Characterization of flagellar propulsion of soft microrobotic sperm in a viscous heterogeneous medium. Front. Robot. AI.

[76] Ji P., Murata-Hori M., Lodish H.F. (2011). Formation of mammalian erythrocytes: Chromatin condensation and enucleation. Trends Cell Biol..

[77] Alapan Y., Yasa O., Schauer O., Giltinan J., Tabak A.F., Sourjik V., Sitti M. (2018). Soft erythrocyte-based bacterial microswimmers for cargo delivery. Sci. Robot..

[78] Gao C., Lin Z., Wang D., Wu Z., Xie H., He Q. (2019). Red blood cell-mimicking micromotor for active photodynamic cancer therapy. ACS Appl. Mater. Interfaces.

[79] Dreyfus R., Baudry J., Roper M., Fermigier M., Stone H.A., Bibette J. (2005). Microscopic artificial swimmers. Nature.

[80] Wu Z., de Ávila B.E.-F., Martín A., Christianson C., Gao W., Thamphiwatana S.K., Escarpa A., He Q., Zhang L., Wang J. (2015). RBC micromotors carrying multiple cargos towards potential theranostic applications. Nanoscale.

[81] Guo J., Agola J.O., Serda R., Franco S., Lei Q., Wang L., Minster J., Croissant J.G., Butler K.S., Zhu W. (2020). Biomimetic rebuilding of multifunctional red blood cells: Modular design using functional components. ACS Nano.

[82] Williams B.J., Anand S.V., Rajagopalan J., Saif M.T.A. (2014). A self-propelled biohybrid swimmer at low Reynolds number. Nat. Commun..

[83] Park S.-J., Gazzola M., Park K.S., Park S., Di Santo V., Blevins E.L., Lind J.U., Campbell P.H., Dauth S., Capulli A.K. (2016). Phototactic guidance of a tissue-engineered soft-robotic ray. Science.

[84] Fu F., Shang L., Chen Z., Yu Y., Zhao Y. (2018). Bioinspired living structural color hydrogels. Sci. Robot..

[85] Chen Z., Fu F., Yu Y., Wang H., Shang Y., Zhao Y. (2019). Cardiomyocytes-actuated Morpho butterfly wings. Adv. Mater..

[86] Shang Y., Chen Z., Fu F., Sun L., Shao C., Jin W., Liu H., Zhao Y. (2019). Cardiomyocyte-driven structural color actuation in anisotropic inverse opals. ACS Nano.

[87] Sun L., Chen Z., Bian F., Zhao Y. (2019). Bioinspired soft robotic caterpillar with cardiomyocyte drivers. Adv. Funct. Mater..

[88] Yang Q., Xu L., Zhong W., Yan Q., Gao Y., Hong W., She Y., Yang G. (2020). Recent advances in motion control of micro/nanomotors. Adv. Intell. Syst..

[89] Lehn J.-M. (1988). Supramolecular chemistry—scope and perspectives molecules, supermolecules, and molecular devices (Nobel Lecture). Angew. Chem. Int. Ed. Engl..

[90] Shao J., Abdelghani M., Shen G., Cao S., Williams D.S., van Hest J.C.M. (2018). Erythrocyte membrane modified Janus polymeric motors for thrombus therapy. ACS Nano.

[91] Hess H., Clemmens J., Qin D., Howard J., Vogel V. (2001). Light-controlled molecular shuttles made from motor proteins carrying cargo on engineered surfaces. Nano Lett..

[92] Ross T.D., Lee H.J., Qu Z.J., Banks R.A., Phillips R., Thomson M. (2019). Controlling organization and forces in active matter through optically defined boundaries. Nature.

[93] Zhang R., Redford S.A., Ruijgrok P.V., Kumar N., Mozaffari A., Zemsky S., Dinner A.R., Vitelli V., Bryant Z., Gardel M.L. (2021). Spatiotemporal control of liquid crystal structure and dynamics through activity patterning. Nat. Mater..

[94] Qu Z.J., Schildknecht D., Shadkhoo S., Amaya E., Jiang J., Lee H.J., Larios D., Yang F., Phillips R., Thomson M. (2021). Persistent fluid flows defined by active matter boundaries. Commun. Phys..

[95] Ahmad R., Kleineberg C., Nasirimarekani V., Su Y.-J., Pozveh S.G., Bae A., Sundmacher K., Bodenschatz E., Guido I., Vidaković-Koch T. (2021). Light-powered reactivation of flagella and contraction of microtubule networks: Toward building an artificial cell. ACS Synth. Biol..

[96] Walter J.M., Greenfield D., Bustamante C., Liphardt J. (2007). Light-powering Escherichia coli with proteorhodopsin. Proc. Natl. Acad. Sci. USA.

[97] Sun Y.Y., Liu Y., Zhang D.M., Zhang H., Jiang J.W., Duan R.M., Xiao J., Xing J.J., Zhang D.F., Dong B. (2019). Calligraphy/painting based on a bioinspired light-diven mcromotor with concentration-dependent motion direction reversal and dynamic swarming behavior. ACS Appl. Mater. Interfaces.

[98] Frangipane G., Dell’Arciprete D., Petracchini S., Maggi C., Saglimbeni F., Bianchi S., Vizsnyiczai G., Bernardini M.L., Di Leonardo R. (2018). Dynamic density shaping of photokinetic E. coli. eLife.

[99] Guillamat P., Ignés-Mullol J., Sagués F. (2016). Control of active liquid crystals with a magnetic field. Proc. Natl. Acad. Sci. USA.

[100] Guillamat P., Ignés-Mullol J., Sagues F. (2017). Taming active turbulence with patterned soft interfaces. Nat. Commun..

[101] Zhang B., Snezhko A., Sokolov A. (2022). Guiding self-assembly of active colloids by temporal modulation of activity. Phys. Rev. Lett..

[102] Sipos O., Nagy K., Di Leonardo R., Galajda P. (2015). Hydrodynamic trapping of swimming bacteria by convex walls. Phys. Rev. Lett..

[103] Bianchi S., Saglimbeni F., Di Leonardo R. (2017). Holographic imaging reveals the mechanism of wall entrapment in swimming bacteria. Phys. Rev. X.

[104] Bianchi S., Saglimbeni F., Frangipane G., Dell’Arciprete D., Di Leonardo R. (2019). 3D dynamics of bacteria wall entrapment at a water–air interface. Soft Matter.

[105] Opathalage A., Norton M.M., Juniper M.P.N., Langeslay B., Aghvami S.A., Fraden S., Dogic Z. (2019). Self-organized dynamics and the transition to turbulence of confined active nematics. Proc. Natl. Acad. Sci. USA.

[106] Hardoüin J., Hughes R., Doostmohammadi A., Laurent J., Lopez-Leon T., Yeomans J.M., Ignés-Mullol J., Sagués F. (2019). Reconfigurable flows and defect landscape of confined active nematics. Commun. Phys..

[107] Li Z.-Y., Zhang D.-Q., Li B. (2021). Formation and propagation of solitonlike defect clusters in confined active nematics with chiral anchoring. Phys. Rev. Res..

[108] Mahmud G., Campbell C.J., Bishop K.J.M., Komarova Y.A., Chaga O.Y., Soh S., Huda S., Kandere-Grzybowska K., Grzybowski B.A. (2009). Directing cell motions on micropatterned ratchets. Nat. Phys..

[109] Hess H., Clemmens J., Matzke C.M., Bachand G.D., Bunker B.C., Vogel V. (2002). Ratchet patterns sort molecular shuttles. Appl. Phys. A.

[110] Doot R.K., Hess H., Vogel V. (2007). Engineered networks of oriented microtubule filaments for directed cargo transport. Soft Matter.

[111] Clemmens J., Hess H., Doot R., Matzke C.M., Bachand G.D., Vogel V. (2004). Motor-protein “roundabouts”: Microtubules moving on kinesin-coated tracks through engineered networks. Lab Chip.

[112] Inoue D., Gutmann G., Nitta T., Kabir A.M.R., Konagaya A., Tokuraku K., Sada K., Hess H., Kakugo A. (2019). Adaptation of patterns of motile filaments under dynamic boundary conditions. ACS Nano.

[113] Thijssen K., Khaladj D.A., Aghvami S.A., Gharbi M.A., Fraden S., Yeomans J.M., Hirst L.S., Shendruk T.N. (2021). Submersed micropatterned structures control active nematic flow, topology, and concentration. Proc. Natl. Acad. Sci. USA.

[114] Clemmens J., Hess H., Howard A.J., Vogel V. (2003). Analysis of microtubule guidance in open microfabricated channels coated with the motor protein kinesin. Langmuir.

[115] Clemmens J., Hess H., Lipscomb R., Hanein Y., Böhringer K.F., Matzke C.M., Bachand G.D., Bunker B.C., Vogel V. (2003). Mechanisms of microtubule guiding on microfabricated kinesin-coated surfaces: Chemical and topographic surface patterns. Langmuir.

[116] Saper G., Tsitkov S., Katira P., Hess H. (2021). Robotic end-to-end fusion of microtubules powered by kinesin. Sci. Robot..

[117] Hess H., Clemmens J., Howard J., Vogel V. (2002). Surface imaging by self-propelled nanoscale probes. Nano Lett..

[118] Hess H., Howard J., Vogel V. (2002). A piconewton forcemeter assembled from microtubules and kinesins. Nano Lett..

[119] Wang H., Pumera M. (2015). Fabrication of micro/nanoscale motors. Chem. Rev..

[120] Park S.J., Park S.-H., Cho S., Kim D.-M., Lee Y., Ko S.Y., Hong Y., Choy H.E., Min J.-J., Park J.-O. (2013). New paradigm for tumor theranostic methodology using bacteria-based microrobot. Sci. Rep..

[121] Zhuang J., Sitti M. (2016). Chemotaxis of bio-hybrid multiple bacteria-driven microswimmers. Sci. Rep..

[122] Park B.-W., Zhuang J., Yasa O., Sitti M. (2017). Multifunctional bacteria-driven microswimmers for targeted active drug delivery. ACS Nano.

[123] Singh A.V., Hosseinidoust Z., Park B.-W., Yasa O., Sitti M. (2017). Microemulsion-based soft bacteria-driven microswimmers for active cargo delivery. ACS Nano.

[124] Liu X.Y., Yuk H., Lin S.T., Parada G.A., Tang T.C., Tham E., de la Fuente-Nunez C., Lu T.K., Zhao X.H. (2018). 3D printing of living responsive materials and devices. Adv. Mater..

[125] Keya J.J., Kabir A.M.R., Inoue D., Sada K., Hess H., Kuzuya A., Kakugo A. (2018). Control of swarming of molecular robots. Sci. Rep..

[126] Keya J.J., Suzuki R., Kabir A.M.R., Inoue D., Asanuma H., Sada K., Hess H., Kuzuya A., Kakugo A. (2018). DNA-assisted swarm control in a biomolecular motor system. Nat. Commun..

[127] Curatolo A.I., Zhou N., Zhao Y., Liu C., Daerr A., Tailleur J., Huang J. (2020). Cooperative pattern formation in multi-component bacterial systems through reciprocal motility regulation. Nat. Phys..

[128] Haeufle D.F.B., Bäuerle T., Steiner J., Bremicker L., Schmitt S., Bechinger C. (2016). External control strategies for self-propelled particles: Optimizing navigational efficiency in the presence of limited resources. Phys. Rev. E.

[129] Qian B., Montiel D., Bregulla A., Cichos F., Yang H. (2013). Harnessing thermal fluctuations for purposeful activities: The manipulation of single micro-swimmers by adaptive photon nudging. Chem. Sci..

[130] Yang Y., Bevan M.A. (2018). Optimal navigation of self-propelled colloids. ACS Nano.

[131] Yang Y., Bevan M.A., Li B. (2020). Micro/nano motor navigation and localization via deep reinforcement learning. Adv. Theory Simul..

[132] Yang Y., Bevan M.A., Li B. (2019). Efficient navigation of colloidal robots in an unknown environment via deep reinforcement learning. Adv. Intell. Syst..

[133] Yang Y., Bevan M.A. (2020). Cargo capture and transport by colloidal swarms. Sci. Adv..

[134] Xu K., Yang Y., Li B. (2021). Brownian cargo capture in mazes via intelligent colloidal microrobot swarms. Adv. Intell. Syst..

[135] David S., Fleischner R. (1966). Fantastic Voyage.

